# Rethinking Anti-Inflammatory Therapy in Alzheimer’s Disease: From Broad Suppression to Stage–State–Space Neuroimmune Reprogramming

**DOI:** 10.3390/cells15131208

**Published:** 2026-07-02

**Authors:** Xiaopu Li, Xingyu Wang, Jiaxing Dou, Jiahui Wang, Feng Xue

**Affiliations:** 1 Hwamei College of Life and Health Sciences, Zhejiang Wanli University, Ningbo 315100, China; elixir1207@163.com (X.L.); 17614756601@163.com (J.D.);; 2 Zhejiang Key Laboratory of Intelligent Food Logistic and Processing, Yuyao Innovation Institute, Zhejiang Wanli University, Ningbo 315400, China

**Keywords:** Alzheimer’s disease, neuroinflammation, microglia, astrocytes, biomarkers, precision therapy, multi-omics, neuroimmune reprogramming

## Abstract

Alzheimer’s Disease (AD) is now understood as a biologically diverse condition, with amyloid and tau pathology evolving within dynamic neuroimmune networks. This challenges the traditional view that AD-related inflammation can be broadly suppressed therapeutically. We review evidence showing that neuroinflammation in AD is stage-dependent, cell-state-specific, spatially organized, and functionally complex. Microglia and astrocytes can aid in plaque containment, debris clearance, synaptic balance, metabolic adaptation, and tissue repair, but may also exacerbate injury through type-I interferon, inflammasome, complement, tumor necrosis factor, and lipid pathways. Many failed anti-inflammatory trials likely stem from mismatches in targets, timing, spatial considerations, pathway redundancy, and biomarker selection, rather than invalidating neuroinflammation as a therapeutic target. Recent single-cell and spatial transcriptomic, proteomic, metabolomic, and network-medicine studies offer a framework for precision intervention by identifying inflammatory endotypes, anatomical niches, and pathway modules. We propose the Stage–State–Space Neuroimmune Reprogramming Model (S3-NRM), aligning AD immunotherapy with disease stage, glial/endotype state, and spatial inflammatory niche, guided by fluid, imaging, and omics biomarkers. Future therapies should selectively suppress harmful immune responses while preserving beneficial glial functions.

## 1. Introduction

Alzheimer’s disease (AD) is the leading cause of dementia worldwide and represents a major and growing global health burden. Contemporary frameworks define AD as a biologically heterogeneous disorder characterized by the interplay of amyloid-β (Aβ) deposition, tau pathology, neurodegeneration, and systemic and central nervous system (CNS) alterations [1,2,3]. Among these processes, neuroinflammation has emerged as a central and genetically supported component of disease pathophysiology. Genome-wide association studies (GWAS) have consistently identified risk loci enriched in myeloid and glial pathways, including genes such as TREM2, CD33, ABI3, and PLCG2, thereby implicating innate immune dysfunction as a core driver of AD rather than a secondary bystander process [4,5,6].

Despite this strong biological rationale, the clinical translation of anti-inflammatory strategies in AD has been largely unsuccessful. Multiple therapeutic programs, including nonsteroidal anti-inflammatory drugs (NSAIDs), cytokine-targeting biologics, kinase inhibitors, and metabolic modulators, have failed to demonstrate robust and reproducible clinical benefit in randomized trials [3,7,8]. This apparent paradox has often been interpreted as evidence that neuroinflammation is not a viable therapeutic target in AD. However, such a conclusion is increasingly difficult to reconcile with genetic, mechanistic, and multi-omics data. A more plausible interpretation is that prior approaches targeted the correct biological domain but were guided by an oversimplified conceptual model—namely, that inflammation in AD represents a uniform and deleterious process that can be mitigated by broad suppression [3,9].

Recent advances fundamentally challenge this assumption. Neuroinflammation in AD is now understood as a dynamic, multicellular network that is temporally evolving, spatially heterogeneous, and functionally ambivalent [10]. Microglia and astrocytes, the principal immune effector cells of the CNS, do not operate as uniformly detrimental entities. Instead, they exhibit context-dependent phenotypes that can either protect neural circuits, through plaque compaction, debris clearance, synaptic support, and metabolic adaptation, or contribute to disease progression via amplification of type-I interferon signaling, inflammasome activation, complement-mediated synapse loss, and tumor necrosis factor (TNF)-driven inflammation [11,12,13,14,15,16,17,18,19]. This duality implies that indiscriminate suppression of inflammatory pathways may inadvertently inhibit protective immune functions while failing to adequately restrain maladaptive ones.

A second major shift in the field arises from the application of high-dimensional multi-omics technologies. Single-cell RNA sequencing, spatial transcriptomics, proteomics, and metabolomics have revealed that glial activation in AD is not binary but instead consists of diverse and coexisting cellular states that vary across brain regions, disease stages, and genetic backgrounds [20,21,22,23,24]. These studies demonstrate that plaque-associated microglia, interferon-responsive populations, lipid-dysregulated states, and complement-enriched astrocytes can coexist within the same brain, often within spatially restricted niches [10]. Importantly, proteomic analyses further indicate that disease-relevant alterations are frequently more pronounced at the protein level than at the transcript level, underscoring the need for integrated, multi-layered analysis of neuroimmune dysfunction [25,26,27].

Together, these insights necessitate a conceptual redefinition of anti-inflammatory therapy in AD. Rather than broadly suppressing inflammatory mediators, emerging evidence supports a strategy of neuroimmune reprogramming, in which therapeutic interventions selectively reshape maladaptive glial trajectories while preserving or restoring beneficial immune functions. In practical terms, this involves enhancing protective processes such as phagocytosis, metabolic fitness, and plaque containment, while dampening pathogenic signaling axes including cGAS–STING/type-I interferon pathways, NLRP3 inflammasome activation, excessive complement signaling, and soluble TNF-driven amplification [18,19,28,29,30,31,32,33].

In this review, we synthesize evidence from human genetics, glial biology, clinical trials, and multi-omics studies to propose a unifying translational framework: the Stage–State–Space Neuroimmune Reprogramming Model (S3-NRM). This model integrates three critical dimensions of AD neuroinflammation: (1) disease stage, which determines reversibility and therapeutic window; (2) cellular state or inflammatory endotype, which defines mechanistic vulnerability; and (3) spatial niche, which captures the anatomical localization of inflammatory processes. By aligning therapeutic strategies with these dimensions and incorporating biomarker-guided patient stratification, S3-NRM provides a rational path toward precision immunotherapy in AD. We argue that this shift, from suppression to reprogramming, may represent the next critical phase in the development of effective disease-modifying therapies.

## 2. Neuroinflammation as a Dynamic Multicellular Network

### 2.1. Microglia as Adaptive but Failure-Prone Immune Hubs

Microglia are not passive “activated cells” but highly plastic, context-dependent immune sentinels whose behavior in AD is shaped by aging, APOE genotype, amyloid burden, tau pathology, metabolic status, and regional circuit environment [4,5]. Foundational studies on disease-associated microglia (DAM) demonstrated that AD-related microglial responses involve a staged transition from homeostatic programs toward plaque-associated states enriched in lipid metabolism, phagocytosis, and innate immune signaling [11,12]. Within this framework, triggering receptor expressed on myeloid cells 2 (TREM2) functions as a central checkpoint that governs microglial activation, clustering around plaques, and metabolic fitness [12,13,14].

Importantly, this transition is not uniformly pathogenic. TREM2-dependent microglial responses promote plaque compaction and limit neuritic dystrophy, whereas impaired TREM2 signaling exacerbates amyloid pathology and neuronal injury [12,13,14]. These findings highlight a critical conceptual limitation of broad anti-inflammatory strategies: early or appropriately directed microglial activation may be protective rather than harmful [34].

**Figure 1:**
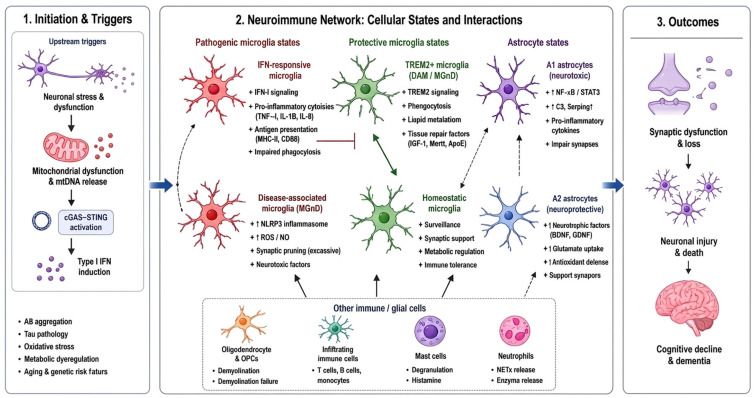
AD neuroinflammation as a dynamic multicellular network. Schematic overview of the cellular and molecular interactions underlying neuroinflammation during AD progression. Upstream pathological triggers, including neuronal stress, mitochondrial dysfunction, and cytosolic danger-associated signals, initiate innate immune activation and inflammatory cascades within the central nervous system. Distinct microglial phenotypes are illustrated, including pro-inflammatory/pathogenic states and protective/homeostatic states, highlighting their dynamic transitions and reciprocal regulation. Astrocyte populations are similarly represented as heterogeneous functional states with either neurotoxic or neuroprotective properties. Additional glial and infiltrating immune cell populations further contribute to the inflammatory microenvironment and intercellular signaling landscape. Communication between neural and immune cells occurs through soluble mediators, direct cell–cell interactions, extracellular vesicles, and metabolic signaling pathways. Persistent imbalance of these neuroimmune networks ultimately promotes synaptic dysfunction, neuronal degeneration, and cognitive decline associated with AD pathology. Solid arrows indicate activation or progression pathways, inhibitory lines indicate suppressive interactions, and dashed arrows represent bidirectional or context-dependent communication. ↑ = increase.

However, adaptive microglial responses can progressively deteriorate into maladaptive states. Increasing evidence from both human tissue and experimental models identifies interferon-responsive, lipid-dysregulated, senescence-like, and synapse-engulfing microglial phenotypes that correlate with chronic neurodegeneration [18,19,31]. Among these, type-I interferon signaling has emerged as a key driver linking innate immune activation to synaptic dysfunction and cognitive decline. Roy et al. demonstrated that coordinated interferon signaling across microglia and neural cells promotes synapse loss and memory impairment in AD models [18,19]. More recent work further connects tau pathology to activation of the cGAS–interferon axis, reinforcing the role of nucleic-acid sensing in driving maladaptive microglial transitions [31] (Figure 1).

In parallel, microglial dysfunction is tightly coupled to metabolic failure. Disruption of metabolic reprogramming impairs microglial energy utilization and phagocytic capacity, thereby limiting their ability to respond adaptively to accumulating pathology [28]. Collectively, these findings support a state-transition model in which microglia shift from protective surveillance and containment toward maladaptive inflammatory amplification when nucleic-acid sensing, metabolic collapse, or chronic stress signaling becomes dominant.

### 2.2. Astrocytes as Reactive Amplifiers and Protective Instructors

Astrocytes are equally dynamic participants in AD neuroinflammation. Although early frameworks proposed a binary A1/A2 classification, accumulating evidence indicates that astrocyte activation exists along a continuum of region-specific and functionally distinct states [35,36]. Activated microglia can induce neurotoxic astrocyte programs characterized by complement activation and inflammatory signaling, thereby amplifying neuronal injury [35].

Mechanistically, neurotoxic astrocytes have been shown to induce neuronal death through the release of saturated lipids, shifting the focus from classical cytokine-mediated toxicity toward lipid-driven metabolic and membrane stress [36]. This observation underscores the importance of astrocyte immunometabolism as a central determinant of neuroinflammatory outcomes.

At the same time, astrocytes possess intrinsic regulatory capacity that can support tissue protection and immune coordination. A notable example is astrocyte-derived interleukin-3 (IL-3), which programs microglia to cluster around amyloid deposits and enhances their ability to limit pathology [37]. This finding is particularly instructive because it demonstrates that beneficial neuroimmune modulation may require instructional signaling, rather than simple suppression of inflammatory pathways (Figure 1).

Thus, astrocytes should not be viewed merely as downstream amplifiers of inflammation but as active regulators capable of both propagating and restraining neuroimmune responses depending on context. This duality further reinforces the need for selective modulation rather than indiscriminate inhibition.

### 2.3. Neuron–Glia Crosstalk as the Core of Network-Level Pathology

A key conceptual advance in the field is the recognition that AD neuroinflammation is fundamentally a networked process emerging from neuron–glia interactions, rather than a cell-autonomous inflammatory response. Neurons undergoing amyloid and tau stress release a range of danger-associated signals, including ATP, lipids, protein aggregates, and mitochondrial components, that are sensed by microglia through receptors such as TREM2, complement receptors, inflammasomes, and nucleic-acid sensors [15,16,17,29].

These signals initiate bidirectional communication loops in which microglia and astrocytes dynamically reshape the extracellular environment through cytokines, complement proteins, lipid mediators, and metabolic substrates. A well-characterized example is complement-dependent synapse elimination: astrocyte-derived complement components such as C1q and C3 tag synapses for removal, while microglia mediate phagocytic pruning, contributing to synaptic loss in AD [15,16,17].

Mitochondrial stress further amplifies this network. Cytosolic mitochondrial DNA (mtDNA) released from stressed neurons activates cyclic GMP–AMP synthase (cGAS), leading to stimulator of interferon genes (STING) signaling and downstream type-I interferon responses [29,30]. This pathway provides a direct mechanistic link between neuronal metabolic dysfunction and innate immune activation.

**Figure 2:**
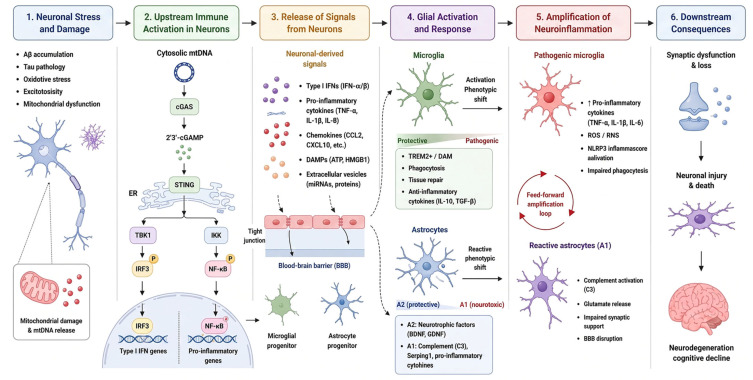
Neuron–glia crosstalk and upstream activation of neuroinflammation in AD. Illustration of the sequential cellular and molecular events driving neuroinflammation during AD progression. Neuronal stressors, including amyloid-β accumulation, tau pathology, oxidative stress, excitotoxicity, and mitochondrial dysfunction, initiate intracellular innate immune signaling pathways in neurons. Mitochondrial damage and cytosolic mitochondrial DNA release activate pattern-recognition pathways, leading to induction of inflammatory transcriptional programs and production of type-I interferons and pro-inflammatory mediators. Neuron-derived cytokines, chemokines, danger-associated molecular patterns (DAMPs), extracellular vesicles, and metabolic signals subsequently influence blood–brain barrier integrity and promote glial activation. Microglia and astrocytes undergo dynamic phenotypic remodeling, transitioning from protective or homeostatic states toward reactive and pathogenic inflammatory states. Activated glial cells further amplify neuroinflammatory signaling through feed-forward interactions, resulting in chronic inflammatory propagation, impaired synaptic support, neuronal injury, and progressive neurodegeneration. ↑ = increase.

Importantly, experimental studies demonstrate that STING signaling is upregulated in both plaque-associated microglia and stressed neurons in AD models, and that pharmacological or genetic inhibition of this pathway reduces gliosis, amyloid burden, tau pathology, synapse loss, and cognitive deficits [32,33]. These findings position the cGAS–STING axis as a central integrator of neuronal stress and neuroimmune dysfunction (Figure 2).

Taken together, these observations support a model in which AD neuroinflammation arises from self-reinforcing neuron–glia feedback loops. The balance between adaptive containment and maladaptive amplification is determined not by the presence of inflammation per se, but by the state, timing, and spatial context of these interactions. This network perspective provides a mechanistic foundation for shifting therapeutic strategies from broad suppression toward targeted neuroimmune reprogramming.

### 2.4. Oxidative Stress and Mitochondrial Dysfunction: Upstream Drivers of Neuroimmune Dysfunction

Beyond the cell-autonomous and intercellular signaling pathways discussed above, a growing body of evidence positions oxidative stress and mitochondrial dysfunction as central upstream drivers that initiate and sustain neuroinflammatory cascades in AD. Mitochondria are not merely passive energy suppliers; they are critical hubs integrating cellular metabolic state, redox balance, and innate immune signaling. In AD, microglial dysfunction has been directly linked to impaired metabolic reprogramming, including altered glycolysis and mitochondrial function [28].

The mechanistic link between mitochondrial dysfunction and neuroinflammation is increasingly well-defined. Stressed or damaged mitochondria release mitochondrial damage-associated molecular patterns (mtDAMPs), including mitochondrial DNA (mtDNA), which are sensed by pattern-recognition receptors on microglia and astrocytes. Cytosolic mtDNA activates cyclic GMP–AMP synthase (cGAS), leading to stimulator of interferon genes (STING) signaling and downstream type-I interferon responses [29,30]. This pathway provides a direct mechanistic link between neuronal metabolic stress and innate immune activation. In AD models, STING signaling is upregulated in both plaque-associated microglia and stressed neurons, and pharmacological or genetic inhibition of this pathway reduces gliosis, amyloid burden, tau pathology, synapse loss, and cognitive deficits [32,33]. More recent work further connects tau pathology to activation of the cGAS–interferon axis, reinforcing the role of nucleic-acid sensing in driving maladaptive microglial transitions [31]. Conversely, mitochondrial stress also activates the NLRP3 inflammasome, promoting IL-1β and IL-18 release and amplifying glial inflammatory responses [38]. This dual activation establishes mitochondria as a critical node linking neuronal metabolic stress to broad neuroimmune activation.

Therapeutically, targeting mitochondrial dysfunction and oxidative stress represents a complementary approach to pathway-specific immunomodulation. Strategies under investigation include mitochondrial antioxidants, agents that enhance mitochondrial biogenesis and quality control (e.g., NAD+ precursors), and modulators of mitophagy. However, the clinical translation of these approaches has been challenging: many antioxidant compounds that showed promise in preclinical models failed in clinical trials, likely due to poor bioavailability, inadequate blood–brain barrier penetration, or intervention at incorrect disease stages. This experience underscores a key rationale for the S3-NRM framework’s emphasis on pathway-specific immunomodulation—namely, that broadly targeting oxidative stress without stage- and state-specific biomarkers may be insufficient, whereas defined immune pathways such as type-I interferon, NLRP3, complement, and TNF offer more tractable, biomarker-guided intervention points with established pharmacologic tools [18,19,38,39].

Importantly, oxidative stress and mitochondrial dysfunction are not mutually exclusive with the S3-NRM framework; rather, they can be conceptually integrated. Stage determines the reversibility of mitochondrial dysfunction—early-stage interventions aimed at restoring mitochondrial health may be more effective than late-stage attempts, as biomarkers such as NfL indicate the extent of ongoing neurodegeneration and the likelihood of reversibility [40,41]. State defines which mitochondrial stress pathways are dominant (e.g., mtDNA-driven cGAS–STING activation versus ROS-driven NLRP3 inflammasome priming), which can be informed by pathway-specific fluid biomarkers [42,43,44]. Space captures regional vulnerabilities, as neurons in high-metabolic-demand regions such as the hippocampus are particularly susceptible to mitochondrial oxidative damage, and imaging modalities such as TSPO-PET can map regional inflammatory niches [45]. Thus, while our review prioritizes immune signaling pathways due to their greater immediate tractability for biomarker-guided clinical intervention, we acknowledge that targeting upstream mitochondrial dysfunction and oxidative stress remains a scientifically valid and potentially complementary strategy that warrants integration into the broader S3-NRM framework.

## 3. Why Conventional Anti-Inflammatory Strategies Have Failed

The failure of conventional anti-inflammatory therapy in AD is best understood not as a single pharmacological limitation but as a systematic translational misalignment between therapeutic strategy and disease biology. Rather than disproving the relevance of neuroinflammation, clinical outcomes suggest that prior approaches were guided by an oversimplified model in which inflammatory activity was treated as a uniform pathological output. AD neuroinflammation is heterogeneous, state-dependent, spatially restricted, and embedded within redundant signaling networks. Here, we synthesize four major and recurring failure modes, target mismatch, temporal and spatial mismatch, network redundancy, and biomarker/trial-design mismatch, that collectively explain the limited success of anti-inflammatory strategies to date [3,7,8,9,46].

### 3.1. Target Mismatch: Suppression Versus Directional Control

Most legacy anti-inflammatory programs in AD assumed that reducing inflammatory signaling would necessarily confer therapeutic benefit (Table 1). However, as discussed above, glial activation in AD includes both protective and pathogenic components. Microglia can support plaque compaction, debris clearance, and metabolic adaptation, while astrocytes can regulate tissue homeostasis and immune coordination [11,12,13,14,37]. Consequently, non-selective suppression risks attenuating beneficial immune functions alongside harmful ones.

The TREM2 pathway illustrates this principle clearly. Genetic and experimental evidence shows that impaired TREM2 signaling compromises microglial responses to amyloid pathology, leading to reduced plaque containment and increased neuronal injury [12,13,14]. Conversely, agonistic targeting of TREM2 can enhance microglial activation and clustering around plaques, at least in preclinical models [13,14]. These findings indicate that the therapeutic problem is not whether to inhibit or activate inflammation globally, but how to direct immune responses toward protective trajectories.

A similar issue arises in TNF-targeting strategies. Broad TNF inhibition with agents such as etanercept has not demonstrated convincing efficacy in AD clinical trials, and compounds such as thalidomide have been limited by poor tolerability [47,48]. However, these outcomes do not negate the role of TNF signaling in AD. Rather, they suggest that indiscriminate inhibition of both soluble and transmembrane TNF may disrupt physiologically relevant immune signaling. More selective approaches, such as inhibition of soluble TNF with XPro1595, attempt to preserve beneficial TNF functions while limiting pathological amplification, highlighting a shift toward functional selectivity rather than global blockade [49,50,51].

### 3.2. Temporal and Spatial Mismatch: Targeting Wrong Process at Wrong Place and Time

Neuroinflammation in AD is not static but evolves qualitatively across disease stages. Early microglial activation may contribute to plaque containment and tissue resilience, whereas later-stage inflammation is often dominated by interferon signaling, complement activation, and inflammasome-driven injury [18,19,32,52]. Interventions that fail to account for this temporal evolution may therefore target processes that are no longer dominant or therapeutically relevant.

The NSAID experience provides a classic example. Epidemiological studies suggested a protective effect of long-term NSAID use, yet randomized clinical trials with agents such as naproxen and celecoxib failed to demonstrate benefit in preventing or delaying AD [7,8]. In retrospect, these discrepancies likely reflect differences in timing, population selection, and underlying mechanisms, with clinical interventions occurring outside the relevant therapeutic window.

Spatial heterogeneity further complicates therapeutic targeting. Neuroinflammatory responses in AD are organized within distinct anatomical niches, including plaque-associated microenvironments, hippocampal circuits, white matter regions, and vascular interfaces [21,22,23]. These niches may harbor distinct glial states and signaling pathways, implying that a systemically administered therapy may fail to achieve sufficient local engagement even if it is mechanistically valid. Moreover, blood–brain barrier (BBB) penetration and regional drug distribution can critically influence therapeutic efficacy, yet are often insufficiently considered in early-stage clinical design.

Together, these observations highlight that effective intervention requires alignment not only with disease stage but also with regional inflammatory context, reinforcing the need for spatially informed therapeutic strategies.

### 3.3. Network Redundancy and Compensatory Escape

A defining feature of AD neuroinflammation is its organization as a redundant and interconnected signaling network. Pathways such as cGAS–STING, type-I interferon signaling, NLRP3 inflammasome activation, TNF signaling, complement cascades, and metabolic stress responses are not independent modules but mutually reinforcing components of a broader inflammatory system [18,32,38,39,53]. As a result, inhibition of a single node may be insufficient to produce durable therapeutic effects due to compensatory activation of parallel pathways.

Clinical and preclinical data support this interpretation. The TREM2 agonist AL002 demonstrated sustained target engagement and pharmacodynamic evidence of microglial activation yet failed to produce meaningful clinical benefit in a phase 2 trial [13,14,54,55]. More fundamentally, one must consider whether sustained TREM2 agonism—rather than simply restoring deficient TREM2 signaling—may have induced receptor desensitization or internalization, thereby paradoxically attenuating the very microglial functions it was intended to enhance. Preclinical evidence indeed suggests that TREM2 agonist antibodies, despite showing agonism in culture models, do not confer therapeutic benefit in AD mouse models and may even worsen outcomes in certain contexts, raising the possibility that pharmacological TREM2 activation is inherently more complex than initially anticipated. This interpretation does not invalidate TREM2 as a therapeutic target, but it shifts the focus from agonism toward alternative approaches such as stabilizing TREM2 expression, enhancing downstream signaling selectivity, or combining TREM2 modulation with parallel pathway inhibition. Similarly, inhibition of stress kinases (e.g., p38α with neflamapimod) or modulation of immunometabolic pathways (e.g., PPARγ with pioglitazone) has not translated into clinical efficacy despite strong mechanistic rationale [56,57,58,59].

These findings underscore a key limitation of single-target monotherapy in a network-driven disease. Without addressing the broader signaling architecture, therapeutic effects may be buffered or negated by compensatory mechanisms. This provides a strong rationale for combination strategies or interventions that target central network hubs rather than isolated pathways.

### 3.4. Biomarker and Trial-Design Mismatch

A further major limitation of past anti-inflammatory trials in AD is the lack of biomarker-driven patient selection and mechanistically aligned endpoints. Many earlier studies were conducted prior to the widespread adoption of amyloid and tau biomarkers, resulting in heterogeneous study populations with uncertain underlying pathology [60,61]. In addition, few trials incorporated biomarkers capable of defining inflammatory endotypes or confirming target engagement.

This lack of biological stratification likely diluted potential treatment effects and contributed to false-negative outcomes. For example, therapies targeting specific inflammatory pathways may only be effective in subsets of patients characterized by corresponding pathway activation, such as interferon-high or complement-high endotypes. Without enrichment for such populations, therapeutic signals may be obscured.

More recent trial designs have begun to address these limitations. The XPro1595 (MINDFuL) study incorporates enrichment for early AD patients with evidence of inflammation and includes exploratory biomarker endpoints to assess pharmacodynamic effects [50,51]. Although definitive efficacy data are still pending, this approach represents a shift toward mechanistically informed clinical design.

In parallel, advances in blood-based biomarkers, including plasma p-tau217, GFAP, sTREM2, and neurofilament light chain (NfL), now enable scalable and minimally invasive assessment of disease biology and treatment response [40,42,43,44]. These tools provide a foundation for adaptive and biomarker-guided trial architectures, which are likely to be essential for future success in neuroimmune-targeted therapies.

## 4. Multi-Omics Evidence and Translational Therapeutic Landscape

### 4.1. Single-Cell and Spatial Data Redefine the Therapeutic Question

A major conceptual shift in AD research has been driven by the transition from bulk tissue analyses to high-dimensional, cell-resolved profiling. Early single-cell transcriptomic studies demonstrated that disease-associated molecular changes are highly cell-type-specific and often obscured in bulk datasets [20]. Subsequent large-scale efforts have expanded this view dramatically. For example, recent multiregion single-nucleus transcriptomic atlases encompassing over one million nuclei across multiple brain regions have revealed distinct neuronal and glial vulnerability patterns that vary by anatomical location and disease stage [21,22,23].

These studies collectively demonstrate that AD neuroinflammation is not a uniform process but consists of diverse and coexisting glial states distributed across spatially defined niches. Plaque-associated microglia, white-matter-associated microglia, interferon-responsive populations, and complement-enriched astrocytes can coexist within the same brain, often in close proximity yet exhibiting distinct transcriptional programs [21,22,23]. Importantly, these states are influenced by genetic background. For instance, APOE genotype has been shown to shape cell-type-specific pathological landscapes, further supporting the need for genotype-aware therapeutic strategies [24].

From a translational perspective, these findings shift the central therapeutic question from “Is inflammation increased?” to “Which inflammatory cell states arise, where, and when?” This reframing has profound implications: effective intervention requires targeting specific cellular states within defined spatial contexts rather than globally suppressing inflammatory mediators. It also underscores the need for spatially informed biomarkers and region-sensitive therapeutic strategies.

### 4.2. Proteomics and Metabolomics Reveal Functional Layers Beyond Transcription

While transcriptomic approaches have provided critical insights into cellular heterogeneity, they do not fully capture functional disease mechanisms. Proteomic studies have revealed that many AD-associated molecular alterations are more pronounced, or even uniquely detectable, at the protein level [25,26]. Large-scale analyses of AD brain tissue and cerebrospinal fluid (CSF) have identified early changes in pathways related to energy metabolism, synaptic function, extracellular matrix remodeling, and glial activation [25].

Importantly, discordance between RNA and protein expression highlights the limitations of relying solely on transcriptomic data. Deep multi-layer analyses demonstrate that protein-level changes often diverge significantly from transcriptional patterns, reflecting post-transcriptional regulation, protein turnover, and pathway activity states [26,27]. These observations reinforce the importance of integrating proteomics into mechanistic and therapeutic studies.

Metabolomics adds an additional dimension by capturing the metabolic state of cells and tissues, which is tightly linked to immune function. Microglial dysfunction in AD has been associated with impaired metabolic reprogramming, including altered glycolysis and mitochondrial function [28]. Moreover, systemic metabolic alterations, such as changes in bile acid profiles, have been linked to cognitive impairment and AD pathology, suggesting interactions between peripheral metabolism, the gut microbiome, and central neuroinflammatory processes [62,63].

Together, proteomic and metabolomic data reveal that AD neuroinflammation is not solely defined by gene expression but is deeply embedded in functional and metabolic networks. These layers are particularly important for therapeutic translation, as they more directly reflect pathway activity and druggable biology.

### 4.3. Network and AI Approaches Enable Rational Combination Strategies

The complexity of AD neuroinflammation necessitates analytical frameworks capable of integrating multi-layered data across cell types, pathways, and spatial contexts. Network medicine and AI-based approaches have emerged as powerful tools for addressing this challenge. By integrating transcriptomic, proteomic, and genetic data, these methods can identify key regulatory nodes, infer pathway interactions, and prioritize therapeutic targets within complex biological systems.

Recent studies using multiscale proteomic modeling have mapped disease-associated protein networks that drive AD pathogenesis, revealing interconnected modules rather than isolated signaling pathways. Building on this, AI-guided approaches have been used to identify BBB-penetrant compounds and to design cell-type-directed combination therapies that correct network-level dysfunction [64].

Notably, preclinical evidence suggests that such combination strategies may outperform single-agent interventions by simultaneously targeting multiple components of the neuroimmune network. This is particularly relevant given the redundancy and compensatory mechanisms described above. Rather than inhibiting a single pathway, effective therapies may need to rebalance entire network states, for example by combining modulation of microglial activation, suppression of maladaptive inflammatory signaling, and restoration of metabolic function.

**Figure 3:**
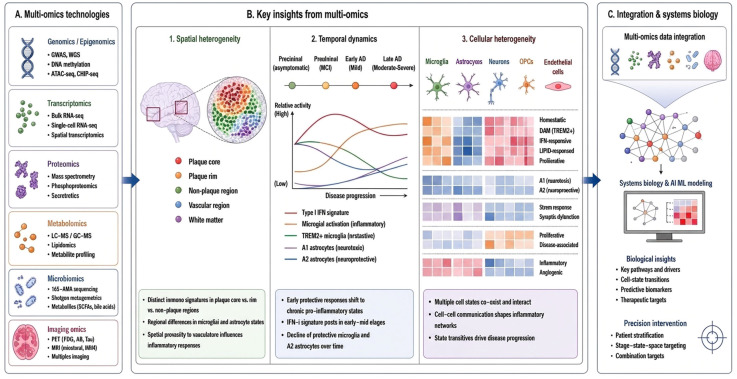
Multi-omics-to-therapy translation pipeline for AD neuroimmune targeting. This schematic illustrates how multi-omics technologies can be integrated to comprehensively characterize the heterogeneous neuroinflammatory landscape of AD. The left panel summarizes major omics platforms commonly applied in AD research, including genomics/epigenomics, transcriptomics, proteomics, metabolomics, microbiome profiling, and imaging-based omics approaches, each providing complementary molecular and cellular information. The central panel highlights three major dimensions of neuroinflammatory heterogeneity revealed by integrated analyses. Spatial heterogeneity analyses identify region-specific inflammatory signatures across plaque cores, plaque rims, vascular-associated regions, white matter, and non-plaque areas. Temporal dynamics analyses depict progressive shifts in inflammatory signaling and glial activation states during disease progression from preclinical stages to advanced AD. Cellular heterogeneity analyses reveal diverse transcriptional and functional states among microglia, astrocytes, neurons, oligodendrocyte lineage cells, and endothelial-associated populations, emphasizing extensive cell-state remodeling and intercellular communication during neurodegeneration. The right panel illustrates systems-level integration of multi-omics datasets using computational biology and artificial intelligence/machine learning frameworks to reconstruct disease networks, identify key regulatory pathways, discover predictive biomarkers, and prioritize therapeutic targets.

From a translational standpoint, these approaches mark a critical transition from empiric drug testing toward mechanism-guided, systems-level therapeutic design (Figure 3). While challenges remain in validating and implementing these strategies clinically, the integration of multi-omics data with network-based modeling provides a feasible path toward precision neuroimmune intervention in AD.

## 5. Biomarker-Guided Precision Therapy

A central implication of the preceding sections is that effective neuroimmune intervention in AD cannot rely on uniform treatment paradigms but must instead be guided by biologically informed patient stratification and longitudinal monitoring. Given the heterogeneity of neuroinflammatory states across patients, disease stages, and anatomical niches, biomarker-guided precision strategies are not optional but essential for therapeutic success [60,61].

A practical framework for biomarker-guided neuroimmune therapy in AD should be layered rather than monolithic, integrating diagnostic confirmation, inflammatory endotyping, and pharmacodynamic monitoring. First, AD biology must be confirmed using established amyloid and tau markers, such as plasma phosphorylated tau (p-tau217) in combination with Aβ42/40 ratios, CSF biomarkers, or amyloid PET imaging [65]. This step ensures that neuroimmune interventions are applied within a biologically defined AD population, avoiding dilution of treatment effects in heterogeneous dementia cohorts.

Second, patients should be stratified according to neuroimmune endotypes, which reflect dominant inflammatory pathways and glial states. Current biomarker evidence already supports this approach, although formal endotype definitions remain under development. Plasma glial fibrillary acidic protein (GFAP) is a sensitive marker of astrocytic reactivity and often rises early in association with amyloid pathology, making it particularly useful for identifying astrocyte-dominant inflammatory states [42]. In parallel, soluble TREM2 (sTREM2) in CSF or plasma reflects microglial activation dynamics and may help identify patients with active myeloid responses who could benefit from microglia-targeted interventions [43,44]. YKL-40 provides a broader measure of glial inflammatory burden, often weighted toward astrocytic activation, and may capture chronic neuroinflammatory states that are less pathway-specific [66,67].

Importantly, these markers should not be interpreted in isolation but rather as components of a multi-dimensional biomarker panel. For example, combining GFAP with sTREM2 may help distinguish astrocyte-dominant versus microglia-dominant inflammatory states, while integration with emerging transcriptomic or proteomic signatures, such as interferon-response or complement activation modules, may further refine endotype classification [42,43,44]. In this context, interferon-related gene or protein signatures may identify patients with cGAS–STING-driven inflammation, whereas complement-related biomarkers may indicate synaptotoxic, complement-mediated pathology, providing mechanistic guidance for targeted intervention.

Third, biomarkers should be used to assess disease stage and therapeutic window. Plasma or CSF NfL serves as a sensitive marker of neuroaxonal injury and can provide insight into the extent of ongoing degeneration and the likelihood of reversibility [40,41]. Elevated NfL levels may indicate advanced or rapidly progressive disease, in which monotherapy targeting a single inflammatory pathway may be insufficient. Conversely, lower NfL levels in early-stage disease may identify a window in which neuroimmune reprogramming is more likely to restore functional homeostasis.

Spatial information represents an additional, often underutilized but fundamentally important dimension. Positron emission tomography (PET) imaging using translocator protein (TSPO) ligands provides regional measures of glial activation, enabling mapping of inflammatory niches within the brain [45]. Although TSPO-PET is constrained by limited cellular specificity and ligand-related polymorphism effects, emerging PET tracers targeting novel markers—such as CSF1R for microglial proliferation, P2X7R for inflammasome-associated activation, and MAO-B for astrocyte reactivity—offer improved potential to distinguish beneficial from pathogenic glial states across different anatomical regions. Complementing PET, magnetic resonance imaging (MRI) provides radiation-free and widely accessible approaches. Diffusion tensor imaging (DTI)-derived free water (FW) has emerged as a sensitive non-invasive biomarker reflecting glial activation and tissue edema, with correlations to plasma glial markers and predictive value for cognitive decline. MR spectroscopy (MRS) enables measurement of neuroinflammatory metabolites such as myo-inositol, while dynamic contrast-enhanced (DCE)-MRI and arterial spin labeling (ASL)-MRI can assess blood–brain barrier permeability and perfusion abnormalities tightly linked to neuroinflammatory processes. Importantly, these imaging modalities can detect the co-existence of neuroprotective and neurodegenerative inflammatory states in different brain regions of the same individual—directly supporting the “Space” dimension of the S3-NRM. Although challenges in specificity, standardization, and clinical implementation remain, the integration of PET- and MRI-based neuroinflammatory markers into multimodal biomarker frameworks is essential for translating spatially informed therapeutic strategies into practice.

Finally, longitudinal biomarker assessment is critical for evaluating target engagement and pharmacodynamic response. Changes in pathway-specific biomarkers, such as interferon signatures, complement proteins, or glial activation markers, can provide early evidence of biological effect, even in the absence of immediate clinical improvement. This is especially important in AD, where clinical endpoints evolve slowly and may not fully capture early therapeutic impact [46,60].

Taken together, these considerations support a shift toward biomarker-integrated trial design, in which enrollment, stratification, and outcome assessment are all guided by mechanistically relevant biomarkers. In the context of the proposed S3-NRM, biomarkers serve as the operational bridge linking disease stage (e.g., via NfL and clinical measures), cellular state (e.g., via GFAP, sTREM2, and pathway signatures), and spatial context (e.g., via imaging modalities). This integrated approach is essential for translating advances in neuroimmune biology into effective and personalized therapeutic strategies for AD.

To translate the above biomarker framework into clinical and trial practice, we propose a three-tiered stratification algorithm that aligns with the S3-NRM dimensions:

Tier 1—Diagnostic confirmation (Stage): All patients should first undergo AD biomarker confirmation using plasma p-tau217 in combination with Aβ42/40 ratio, or CSF biomarkers/amyloid PET when available [60,65]. Plasma NfL provides additional staging information regarding the extent of ongoing neuroaxonal injury [40,41]. This tier is widely accessible via commercial labs (e.g., Quest, Labcorp, C2N Diagnostics) at a cost of approximately $200–500 per panel and is already integrated into routine clinical workflows in specialized memory clinics.

Tier 2—Inflammatory endotyping (State): Patients should then be stratified by neuroimmune endotype using a multiplex plasma panel that includes GFAP (astrocytic reactivity), sTREM2 (microglial activation), YKL-40 (glial inflammatory burden), and, where available, pathway-specific signatures such as interferon-response or complement activation modules [42,43,44,66,67]. This panel can be run on widely available platforms such as SIMOA or ELISA, with costs ranging from $300–800 per patient depending on the number of analytes. Importantly, these assays are already offered by several commercial biomarker laboratories and are increasingly adopted in clinical trial settings, though broader clinical adoption will require further standardization and regulatory clearance.

Tier 3—Spatial mapping (Space): For trials where anatomical localization of inflammation is mechanistically central, TSPO-PET imaging should be considered. Current TSPO-PET ligands such as [^11^C]PBR28 and [^18^F]DPA-714 are available at specialized academic PET centers but remain costly ($2,000–5,000 per scan) and are limited by ligand-related polymorphism effects and cellular specificity constraints [45]. Emerging PET tracers targeting CSF1R, P2X7R, and MAO-B may offer improved specificity in the future but are not yet clinically available. Therefore, in the near term, spatial information is best reserved for exploratory mechanistic trials rather than routine stratification.

Regulatory and implementation considerations: The proposed biomarker algorithm faces three major barriers to widespread implementation. First, standardization—plasma biomarker assays vary across platforms and lack unified cutoffs for endotype classification. Second, regulatory validation—while p-tau217 and Aβ42/40 have received regulatory clearance (e.g., FDA clearance for certain AD blood tests), neuroinflammatory markers such as GFAP, sTREM2, and YKL-40 have not yet been formally qualified as companion diagnostics for therapy selection [65]. Third, cost and accessibility—the combined cost of Tier 1+2 panels ($500–1300) is manageable for clinical trials but remains prohibitive for routine primary care use in many health systems. We acknowledge these limitations and emphasize that the proposed framework is intended for biomarker-enriched clinical trials in the near term, with gradual translation to clinical practice as assays become standardized, regulatory pathways are established, and costs decline through economies of scale and technological advances.

## 6. Stage–State–Space Neuroimmune Reprogramming Model: Integrative Framework and Translational Implications

**Figure 4:**
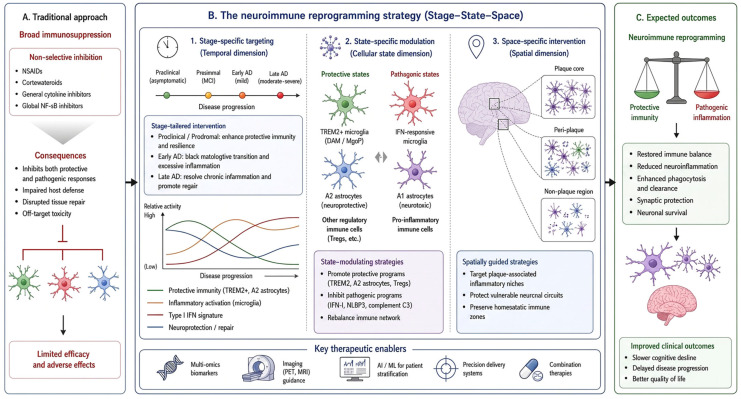
Stage–State–Space Neuroimmune Reprogramming Model (S3-NRM) for AD. This schematic presents the Stage–State–Space Neuroimmune Reprogramming Model (S3-NRM) for AD, contrasting it with traditional broad immunosuppression strategies. Traditional approaches, including NSAIDs, corticosteroids, and global NF-κB inhibitors, employ non-selective inhibition that suppresses both protective and pathogenic immune responses. This leads to impaired host defense, disrupted tissue repair, off-target toxicity, and ultimately limited efficacy. In contrast, the S3-NRM integrates three key dimensions for precision therapy: Stage-specific targeting along the temporal axis of AD progression—from preclinical to late-stage disease—enables stage-tailored interventions, such as enhancing protective immunity in early stages and resolving chronic inflammation in late stages. State-specific modulation targets distinct cellular immune states, promoting protective programs while inhibiting pathogenic ones to rebalance the neuroimmune network. Space-specific intervention focuses on spatially distinct brain regions, such as plaque-associated inflammatory niches and peri-plaque areas, to precisely modulate neuroimmune activity. This integrative model aims to restore immune balance, reduce neuroinflammation, enhance synaptic plasticity and neuronal survival, and ultimately improve clinical outcomes by slowing cognitive decline and delaying disease progression.

We now integrate the preceding biological, clinical, and multi-omics evidence into a unified translational framework, termed the S3-NRM. The central premise of this model is that effective neuroimmune intervention in AD requires simultaneous alignment across three orthogonal but interacting dimensions: disease stage, cellular state (neuroimmune endotype), and spatial inflammatory niche [21,22,23,42,43,44,61,62,63,64]. Unlike traditional linear models that dichotomize inflammation into “early” versus “late” phases, S3-NRM explicitly incorporates the multidimensional heterogeneity revealed by recent human data (Figure 4).

### 6.1. Stage: Defining Therapeutic Window and Directional Logic

The stage dimension captures the temporal evolution of AD and determines both the reversibility of pathology and the appropriate direction of immune modulation. In preclinical or prodromal stages, microglial activation may still retain adaptive functions, including plaque containment, debris clearance, and metabolic support. At this stage, therapeutic strategies may prioritize preservation or enhancement of protective immune responses, while preventing early maladaptive signaling such as excessive nucleic-acid sensing or inflammasome priming [18,32].

In mild symptomatic disease, neuroinflammatory networks often become more complex, with coexistence of adaptive and maladaptive glial states. Here, therapeutic strategies may require selective reprogramming, combining suppression of chronic inflammatory amplifiers (e.g., type-I interferon, complement, NLRP3) with restoration of beneficial microglial and astrocytic functions [10,38]. In later stages, when neurodegeneration is extensive and biomarkers such as NfL are markedly elevated, the potential for reversing established pathology may be limited [40,41]. In such contexts, neuroimmune interventions may need to be integrated with supportive or combinatorial approaches rather than relying on single-pathway modulation.

Thus, stage does not merely indicate disease severity but defines the therapeutic window and directional logic—whether to enhance, suppress, or rebalance immune responses.

### 6.2. State: Targeting Neuroimmune Endotypes Rather than Generic Inflammation

The state dimension reflects the heterogeneity of glial activation and inflammatory pathways within AD. As demonstrated by multi-omics studies, microglia and astrocytes adopt diverse phenotypes, including TREM2-dependent plaque-associated states, interferon-responsive programs, complement-enriched synaptotoxic states, and metabolically impaired or senescence-like phenotypes [20,21,22,23]. These states are not interchangeable and may require distinct therapeutic strategies.

Within the S3-NRM framework, patients can be conceptualized as belonging to different neuroimmune endotypes, such as: (1) TREM2-low or microglial dysfunction states, characterized by impaired sensing and phagocytosis. (2) Interferon-high states, associated with cGAS–STING activation and synaptic injury. (3) Complement-high states, linked to synapse pruning and circuit loss. (4) Immunometabolic collapse states, involving impaired glial energy metabolism.

Each of these endotypes implies a different therapeutic direction. For example, TREM2 agonism may be beneficial in microglial dysfunction states, whereas inhibition of cGAS–STING or interferon signaling may be more appropriate in interferon-dominant states. Similarly, complement-modulating strategies may be most effective in patients with evidence of complement-mediated synaptotoxicity.

Importantly, these endotypes are not mutually exclusive and may coexist within the same patient. Therefore, the goal is not rigid classification but dominant pathway identification, enabling prioritization of therapeutic targets based on the most influential pathological processes.

To operationalize this prioritization in the setting of coexisting endotypes—which is likely the norm in late-stage AD—we propose a hierarchical decision framework based on three criteria: pathogenicity, reversibility, and therapeutic accessibility. First, pathways with direct mechanistic links to ongoing neuronal injury and cognitive decline, such as type-I interferon-driven synaptic dysfunction or complement-mediated synapse loss, should be prioritized over those with more indirect or compensatory effects [18,19,38,39]. Second, pathways that retain reversibility in the current disease stage should take precedence over those that are likely exhausted or fixed—for example, immunometabolic dysfunction may be more amenable to correction in earlier stages than in end-stage neurodegeneration [28,41]. Third, pathways for which validated pharmacologic tools or biomarker readouts already exist should be prioritized over those requiring de novo development [50,51,54,55]. Notably, these criteria are not intended to reduce the S3-NRM to a rigid algorithm, but rather to provide clinicians and trialists with a structured rationale for target selection when multiple inflammatory pathways are concurrently active. In practice, this prioritization may guide the selection of lead therapeutic targets for combination regimens, where a primary pathway is addressed first or at the highest dose, while secondary pathways are co-targeted with lower-intensity or adjunctive interventions. We acknowledge that prospective validation of this prioritization framework in biomarker-enriched clinical trials remains necessary, and emphasize that S3-NRM is a flexible heuristic—not a categorical classification system—for navigating the graded and continuous nature of neuroimmune dysfunction in AD.

### 6.3. Space: Integrating Anatomical Context and Inflammatory Niches

The space dimension captures the spatial organization of neuroinflammation within the brain. AD pathology is not uniformly distributed but is concentrated within specific anatomical niches, including plaque-associated microenvironments, hippocampal circuits, white matter tracts, and vascular interfaces [21,22,23]. These regions differ not only in cellular composition but also in dominant inflammatory pathways and glial states.

Spatial context influences both disease progression and therapeutic response. For example, plaque-associated microglia may be enriched in TREM2-dependent programs, whereas white matter regions may exhibit distinct inflammatory profiles linked to myelin and oligodendrocyte biology. Similarly, hippocampal circuits may be particularly vulnerable to interferon-driven synaptic dysfunction.

Incorporating spatial information into therapeutic design therefore enables region-specific targeting and interpretation of treatment effects. Imaging modalities such as TSPO-PET, as well as emerging spatial transcriptomic and proteomic approaches, provide tools for mapping these inflammatory niches in vivo or ex vivo [45]. Within the S3-NRM framework, spatial information is not an optional refinement but a core determinant of therapeutic alignment.

### 6.4. Integration: From Suppression to Multidimensional Neuroimmune Reprogramming

The defining feature of S3-NRM is the integration of stage, state, and space into a unified therapeutic logic. Rather than applying uniform anti-inflammatory strategies, interventions are tailored to: (1) The stage-dependent therapeutic window. (2) The dominant neuroimmune endotype. (3) The relevant spatial inflammatory niche.

This multidimensional alignment enables a shift from indiscriminate suppression toward selective neuroimmune reprogramming, in which maladaptive pathways are attenuated while beneficial immune functions are preserved or restored.

In practical terms, this approach supports several translational principles. First, combination therapy becomes a rational necessity rather than an optional escalation strategy, given the coexistence of multiple pathways and states within individual patients [64]. Second, biomarker-guided stratification is required to match therapies to endotypes and monitor target engagement, as outlined in Section 5 [42,43,44,60,61]. Third, adaptive trial designs that incorporate longitudinal biomarker feedback may be better suited to capture dynamic changes in neuroimmune states than traditional fixed designs [46].

Finally, S3-NRM provides a framework for interpreting past clinical failures. Interventions such as TREM2 agonists, TNF inhibitors, or metabolic modulators may have failed not because their targets were irrelevant, but because they were applied without sufficient alignment to stage, state, and spatial context [47,49,50,51,68,69,70,71]. By contrast, future strategies that incorporate this multidimensional alignment are more likely to achieve meaningful and reproducible clinical effects.

While S3-NRM is proposed as a conceptual framework rather than a rigid algorithmic tool, its translational utility can be enhanced by operationalizing the relative weighting of its three dimensions in clinical decision-making. One practical approach is to prioritize dimensions based on data availability and therapeutic context: Stage should anchor initial patient selection, as it defines the fundamental reversibility window and determines whether enhancement or suppression of immune responses is appropriate [40,41]. State should guide target selection, as it identifies the dominant inflammatory pathway (e.g., interferon-high versus complement-high) that is most amenable to pharmacologic intervention [20,21,22,23]. Space should inform regional engagement strategies and interpret regional variations in treatment response [45]. In trial settings, these dimensions can be integrated through hierarchical biomarker algorithms: for example, NfL and clinical staging define Stage; plasma GFAP, sTREM2, and pathway-specific signatures define State; and TSPO-PET or emerging spatial biomarkers define Space [42,43,44,45]. We acknowledge that the precise weighting of these dimensions requires prospective validation in biomarker-enriched clinical trials. However, the value of S3-NRM lies not in offering a fixed formula, but in providing a structured framework that forces explicit consideration of all three dimensions—a step that has been largely absent in previous anti-inflammatory trials. This framing, we believe, transforms S3-NRM from a descriptive synthesis into a prescriptive tool for trial design and patient stratification.

## 7. Discussion

The S3-NRM provides a unifying framework for interpreting both the biological complexity of AD neuroinflammation and the repeated failure of conventional anti-inflammatory strategies. Rather than viewing these failures as evidence against neuroinflammation as a therapeutic target, S3-NRM reframes them as failures of therapeutic alignment—where interventions were not matched to the appropriate disease stage, neuroimmune state, or spatial context [3,46].

This perspective helps reinterpret several prominent clinical outcomes. The lack of efficacy observed with TREM2 agonism (e.g., AL002) may not indicate that microglial activation is irrelevant, but rather that enhancing upstream sensing without concurrent modulation of downstream maladaptive pathways is insufficient, particularly in heterogeneous patient populations [13,14,54,55]. Similarly, the limited success of broad TNF inhibition suggests that non-selective cytokine suppression may disrupt physiologically beneficial signaling while failing to adequately suppress chronic inflammatory amplification [47,48]. Negative results from immunometabolic or kinase-targeting approaches, such as pioglitazone and neflamapimod, further underscore that diffuse pathway modulation cannot reliably overcome network redundancy when applied without biological stratification [56,57,58,59].

A key implication of this reinterpretation is that therapeutic success in AD neuroinflammation is likely contingent on directional specificity rather than pathway inhibition alone. Interventions must be designed not simply to reduce inflammatory output, but to reshape immune trajectories—enhancing protective functions while selectively suppressing maladaptive signaling. This distinction is critical because it shifts the therapeutic objective from “less inflammation” to “better-organized inflammation”, a concept more consistent with the dual roles of glial cells in CNS homeostasis and disease.

The S3-NRM framework also highlights the importance of integrating multi-omics data into translational decision-making. Single-cell and spatial transcriptomic studies define cellular states and anatomical niches, while proteomic and metabolomic analyses capture functional pathway activity and systemic interactions [21,22,23,24,25,26,27,62,63]. However, these datasets are not inherently actionable without computational integration. Network-based and AI-assisted approaches therefore play a critical role in translating complex biological data into tractable therapeutic hypotheses, including the identification of combinatorial strategies that target multiple nodes within neuroimmune networks [64].

Indeed, one of the most important translational implications of S3-NRM is the need to move beyond single-agent strategies. Given the redundancy and interconnectivity of inflammatory pathways, combination therapy is likely to be required to achieve durable reprogramming of neuroimmune states. Emerging preclinical studies demonstrating cell-type-directed, network-correcting combination therapies provide proof-of-principle that such approaches can outperform monotherapies in complex neurodegenerative systems. Importantly, these strategies do not merely add multiple agents but are designed to coordinate effects across distinct cell types and pathways, aligning with the multidimensional nature of AD pathology.

Another critical implication concerns clinical trial design. Traditional trial paradigms, which enroll heterogeneous patient populations and rely primarily on cognitive endpoints, are poorly suited to evaluating neuroimmune-targeted therapies. In contrast, S3-NRM supports the use of biomarker-enriched cohorts, mechanistically aligned endpoints, and adaptive trial designs that incorporate longitudinal assessment of target engagement and pathway modulation [43,44,46,60,61]. Such designs are more likely to detect biologically meaningful effects, even when clinical outcomes evolve slowly.

Importantly, S3-NRM should not be interpreted as a rigid classification system but as a flexible and iterative framework. Neuroimmune states are dynamic and may evolve during disease progression or in response to therapy. Therefore, longitudinal monitoring and adaptive therapeutic adjustment are essential components of this approach. This perspective aligns with broader trends in precision medicine, where treatment strategies are continuously refined based on real-time biological feedback.

Despite these advances, several challenges remain. First, the definition and validation of neuroimmune endotypes require further standardization, particularly in the context of clinical biomarker development. Second, the integration of spatial information into routine clinical practice remains technically and logistically challenging. Third, the safety implications of modulating immune pathways, particularly those involved in host defense, must be carefully considered, especially for targets such as cGAS–STING and inflammasome signaling. Finally, translating multi-omics insights into scalable and cost-effective clinical tools remains a significant barrier.

Nevertheless, the convergence of genetic, mechanistic, and multi-omics evidence strongly supports a transition from broad anti-inflammatory suppression to precision neuroimmune reprogramming. By aligning therapeutic strategies with disease stage, neuroimmune state, and spatial context, S3-NRM provides a coherent framework for overcoming past translational failures and guiding the next generation of AD immunotherapies.

An additional critical consideration for the clinical translation of neuroimmune reprogramming strategies is therapeutic safety, particularly in the elderly AD population. Many of the targets advocated in this review—including cGAS-STING, NLRP3 inflammasome, complement components, and TNF—play essential roles in host defense against pathogens, CNS immune surveillance, and tissue homeostasis [53,71]. Chronic pharmacological inhibition of these pathways carries inherent risks of immunosuppression, including increased susceptibility to systemic infections, impaired CNS clearance of pathogens, and potential dysregulation of physiological immune functions such as synaptic pruning and debris clearance [18,19,39,71]. Indeed, safety concerns have been a major contributor to the clinical failure or limitation of several neuroimmune-targeted agents, including tolerability issues with thalidomide and ARIA-like MRI changes observed with TREM2 agonist AL002 [48,54,55,68]. These experiences underscore that selective modulation—rather than complete blockade—of inflammatory pathways is essential, and that the therapeutic window for neuroimmune intervention may be narrower than previously appreciated. Within the S3-NRM framework, safety considerations should inform endotype prioritization and therapeutic design: for example, intermittent dosing schedules, CNS-restricted targeting, or pathway-selective inhibition (e.g., soluble TNF neutralization rather than broad TNF blockade) may offer improved risk–benefit profiles [49,50,51]. We emphasize that future neuroimmune trials must incorporate rigorous safety monitoring, including surveillance for infectious complications and longitudinal assessment of immune competence, alongside efficacy endpoints. Ultimately, the risk–benefit calculus of any neuroimmune reprogramming strategy will depend on disease stage, baseline immune status, and the availability of biomarkers to identify patients most likely to benefit—and least likely to experience harm—from targeted.

## 8. Future Perspectives

The next phase of AD immunotherapy should, in our opinion, prioritize six directions. First, stage-specific interventional timing must be built into trial design from the start. Second, biomarker-defined inflammatory endotypes should replace symptom-only enrollment. Third, spatial readouts, whether through PET, advanced MRI, or spatially informed plasma/CSF surrogates, should be incorporated whenever anatomically localized biology is central to mechanism. Fourth, adaptive platform or umbrella trial structures are likely to outperform serial single-agent trials in this heterogeneous field. Fifth, BBB-shuttled biologics and CNS-optimized small molecules deserve special priority because several attractive targets still suffer from delivery constraints. Sixth, combination regimens, including pairing neuroimmune reprogramming with anti-amyloid or anti-tau backbones, should be explored explicitly rather than postponed.

A final and underappreciated priority is endpoint redesign. Traditional cognitive outcomes remain necessary, but early-phase neuroimmune trials should also establish go/no-go logic using target engagement, pathway-responsive fluid biomarkers, and trajectory-sensitive composite endpoints. Exploratory biomarker-rich trial designs are not a luxury in AD; they are a prerequisite for avoiding another decade of false negatives caused by biological imprecision.

## 9. Conclusions

AD neuroinflammation should no longer be conceptualized as a nonspecific excess of inflammatory mediators awaiting generic suppression. It is a dynamic, multicellular, spatially structured and mechanistically layered network in which microglia and astrocytes can protect, adapt, fail, and become toxic in different combinations over time. The major clinical failures of anti-inflammatory therapy are therefore better understood as failures of therapeutic alignment than as refutations of neuroimmune biology. We conclude that the most rational path forward is a transition from broad anti-inflammatory suppression to Stage–State–Space Neuroimmune Reprogramming, anchored in multi-omics, biomarker-guided patient selection, and mechanism-responsive trial design. If the field adopts this framework, neuroinflammation may become not the graveyard of AD therapeutics, but one of its most promising combination frontiers.

## Figures and Tables

**Table 1:** Selected neuroinflammation-directed therapeutic strategies in AD.

Strategy	Mechanistic Goal	Target(s)	Agent(s)	Clinical Stage	Advantages	Main Limitations
TREM2 agonism	Reinforce microglial sensing, clustering, plaque containment, and metabolic fitness	TREM2/DAP12 axis	AL002; next-generation BBB-shuttled TREM2 agonists	AL002: Phase 2 completed, negative; next-generation agents: preclinical	Genetically validated target; directly addresses microglial dysfunction	Target engagement may not equal clinical benefit; context dependence; ARIA-like MRI changes reported with AL002
Selective soluble TNF neutralization	Reduce maladaptive TNF while preserving useful TNF signaling	Soluble TNF	XPro1595	Phase 2 ongoing/open-label extension	Mechanistically more selective than broad TNF blockade; biomarker-enriched design	Human efficacy not yet established; immune safety and treatment window remain open questions
Broad TNF inhibition	Lower inflammatory cytokine tone	Soluble and transmembrane TNF	Etanercept; thalidomide	Etanercept: Phase 2 completed; thalidomide: early clinical failure	Straightforward mechanism; historical proof of concept for pathway relevance	Weak CNS penetration/poor tolerability; risk of blocking beneficial immune signaling
Innate neuroimmune kinase/mast-cell modulation	Restrain microglia/mast-cell activation and neuroimmune crosstalk	KIT/Lyn/Fyn and related kinases	Masitinib	Phase 3 signal reported; confirmatory public development ongoing/unspecified	Orally available; broader neuroimmune mechanism	Mechanism is pleiotropic; confirmatory evidence still needed
Anti-inflammatory/immunometabolic modulation	Reduce NF-κB/ERK-linked inflammatory tone and insulin-resistance signaling	NF-κB/ERK–metabolic interface	Bezisterim (NE3107)	Phase 3 completed; efficacy interpretation uncertain	Oral small molecule; targets inflammation–metabolism interface	Public efficacy dataset remains difficult to interpret; mechanism is broad
Stress-kinase modulation	Limit synaptotoxic stress signaling and inflammatory kinase activation	p38α MAPK	Neflamapimod	Phase 2 completed, negative in mild AD	Brain-penetrant small molecule; mechanistically plausible	Clinical benefit not shown; may be insufficient as monotherapy
Inflammasome inhibition	Restrain IL-1β/IL-18 releases and pyroptotic amplification	NLRP3 inflammasome	Dapansutrile (OLT1177); MCC950	AD-specific development: preclinical/unspecified	Strong preclinical rationale; directly blocks maladaptive innate amplification	Translational AD biomarker package unclear; chronic safety and BBB considerations unresolved
Nucleic-acid sensing blockade	Interrupt mtDNA/cGAS–STING–IFN inflammatory loop	cGAS–STING	H-151; C-176; future cGAS/STING inhibitors	Preclinical	Strong mechanistic linkage to IFN, NLRP3, synapse loss, and tau/Aβ stress	Potential host-defense liabilities; no established AD human program yet
Complement modulation	Reduce complement-dependent synapse loss and glial inflammatory crosstalk	C1q/C3/C5aR1	PMX205; anti-C1q concepts	Preclinical/AD-specific clinical stage unspecified	Addresses synapse-centric pathology; spatially relevant to plaque niches	Complement biology is context dependent; risk of over-suppressing homeostatic pruning/defense
Microglial reset or replacement	Transiently deplete or reprogram maladaptive microglia to allow repopulation/reset	CSF1R	PLX3397; PLX5622	Preclinical	Conceptually attractive for severe microgliopathy states	Blunt microglial depletion may remove beneficial cells; human AD translation immature
Immunometabolic re-tuning	Improve glial bioenergetics, insulin signaling, and inflammatory–metabolic coupling	PPARγ and related pathways	Pioglitazone	Phase 3 completed, negative	Safe legacy pharmacology; strong systems rationale	Timing likely critical; effect too diffuse or too weak for established disease

## Data Availability

No new data were created or analyzed in this study. Data sharing is not applicable to this article.

## Abbreviations

The following abbreviations are used in this manuscript:

AD	Alzheimer’s Disease
Aβ	Amyloid-β
NFTs	Intraneuronal neurofibrillary tangles
CNS	Central nervous system
GWAS	Genome-wide association studies
NLRP3	NOD-like Receptor Family Pyrin Domain Containing 3
TNF	Tumor necrosis factor
APP	Amyloid precursor protein
TREM2	Triggering Receptor Expressed on Myeloid Cells 2
MG	Microglia
AS	Astrocyte
NSAIDs	Non-Steroidal Anti-Inflammatory Drugs
mtDNA	Mitochondrial DNA
BBB	Blood–Brain Barrier
NfL	Neurofilament light chain
GFAP	Glial fibrillary acidic protein
sTREM2	Soluble TREM2
PET	Positron emission tomography
TSPO	Translocator protein
IFN-I	Type-I interferon
CSF	Cerebrospinal fluid

## References

[1] Alzheimer’s Association (2025). 2025 Alzheimer’s disease facts and figures. Alzheimer’s Dement..

[2] Scheltens P., De Strooper B., Kivipelto M., Holstege H., Chetelat G., Teunissen C.E., Cummings J., van der Flier W.M. (2021). Alzheimer’s disease. Lancet.

[3] Cummings J.L., Zhou Y., Lee G., Zhong K., Fonseca J., Leisgang-Osse A.M., Cheng F. (2025). Alzheimer’s disease drug development pipeline: 2025. Alzheimer’s Dement..

[4] Lambert J.C., Ibrahim-Verbaas C.A., Harold D., Naj A.C., Sims R., Bellenguez C., DeStafano A.L., Bis J.C., Beecham G.W., Grenier-Boley B. (2013). Meta-analysis of 74,046 individuals identifies 11 new susceptibility loci for Alzheimer’s disease. Nat. Genet..

[5] Sims R., van der Lee S.J., Naj A.C., Bellenguez C., Badarinarayan N., Jakobsdottir J., Kunkle B.W., Boland A., Raybould R., Bis J.C. (2017). Rare coding variants in PLCG2, ABI3, and TREM2 implicate microglial-mediated innate immunity in Alzheimer’s disease. Nat. Genet..

[6] Wang X., Rong Z., Xue F. (2026). Multi-Dimensional Transcriptomics Reveals the Prominent Role of Neuroinflammation in Alzheimer’s Disease. Int. J. Mol. Sci..

[7] Group A.R. (2007). Naproxen and celecoxib do not prevent AD in early results from a randomized controlled trial. Neurology.

[8] Alzheimer’s Disease Anti-inflammatory Prevention Trial Research Group (2013). Results of a follow-up study to the randomized Alzheimer’s Disease Anti-inflammatory Prevention Trial (ADAPT). Alzheimer’s Dement..

[9] Heneka M.T., Carson M.J., El Khoury J., Landreth G.E., Brosseron F., Feinstein D.L., Jacobs A.H., Wyss-Coray T., Vitorica J., Ransohoff R.M. (2015). Neuroinflammation in Alzheimer’s disease. Lancet Neurol..

[10] Zhou Y., Huang Y., Fan Y., Xue F. (2025). Co-regulation of microglial subgroups in Alzheimer’s amyloid pathology: Implications for diagnosis and drug development. PLoS ONE.

[11] Keren-Shaul H., Spinrad A., Weiner A., Matcovitch-Natan O., Dvir-Szternfeld R., Ulland T.K., David E., Baruch K., Lara-Astaiso D., Toth B. (2017). A Unique Microglia Type Associated with Restricting Development of Alzheimer’s Disease. Cell.

[12] Ulland T.K., Song W.M., Huang S.C., Ulrich J.D., Sergushichev A., Beatty W.L., Loboda A.A., Zhou Y., Cairns N.J., Kambal A. (2017). TREM2 Maintains Microglial Metabolic Fitness in Alzheimer’s Disease. Cell.

[13] Wang S., Mustafa M., Yuede C.M., Salazar S.V., Kong P., Long H., Ward M., Siddiqui O., Paul R., Gilfillan S. (2020). Anti-human TREM2 induces microglia proliferation and reduces pathology in an Alzheimer’s disease model. J. Exp. Med..

[14] Long H., Simmons A., Mayorga A., Burgess B., Nguyen T., Budda B., Rychkova A., Rhinn H., Tassi I., Ward M. (2024). Preclinical and first-in-human evaluation of AL002, a novel TREM2 agonistic antibody for Alzheimer’s disease. Alzheimer’s Res. Ther..

[15] Lian H., Litvinchuk A., Chiang A.C., Aithmitti N., Jankowsky J.L., Zheng H. (2016). Astrocyte-Microglia Cross Talk through Complement Activation Modulates Amyloid Pathology in Mouse Models of Alzheimer’s Disease. J. Neurosci..

[16] Hong S., Beja-Glasser V.F., Nfonoyim B.M., Frouin A., Li S., Ramakrishnan S., Merry K.M., Shi Q., Rosenthal A., Barres B.A. (2016). Complement and microglia mediate early synapse loss in Alzheimer mouse models. Science.

[17] Dejanovic B., Wu T., Tsai M.C., Graykowski D., Gandham V.D., Rose C.M., Bakalarski C.E., Ngu H., Wang Y., Pandey S. (2022). Complement C1q-dependent excitatory and inhibitory synapse elimination by astrocytes and microglia in Alzheimer’s disease mouse models. Nat. Aging.

[18] Roy E.R., Wang B., Wan Y.W., Chiu G., Cole A., Yin Z., Propson N.E., Xu Y., Jankowsky J.L., Liu Z. (2020). Type I interferon response drives neuroinflammation and synapse loss in Alzheimer disease. J. Clin. Investig..

[19] Roy E.R., Chiu G., Li S., Propson N.E., Kanchi R., Wang B., Coarfa C., Zheng H., Cao W. (2022). Concerted type I interferon signaling in microglia and neural cells promotes memory impairment associated with amyloid beta plaques. Immunity.

[20] Mathys H., Davila-Velderrain J., Peng Z., Gao F., Mohammadi S., Young J.Z., Menon M., He L., Abdurrob F., Jiang X. (2019). Single-cell transcriptomic analysis of Alzheimer’s disease. Nature.

[21] Chen W.T., Lu A., Craessaerts K., Pavie B., Sala Frigerio C., Corthout N., Qian X., Lalakova J., Kuhnemund M., Voytyuk I. (2020). Spatial Transcriptomics and In Situ Sequencing to Study Alzheimer’s Disease. Cell.

[22] Mathys H., Boix C.A., Akay L.A., Xia Z., Davila-Velderrain J., Ng A.P., Jiang X., Abdelhady G., Galani K., Mantero J. (2024). Single-cell multiregion dissection of Alzheimer’s disease. Nature.

[23] Miyoshi E., Morabito S., Henningfield C.M., Das S., Rahimzadeh N., Shabestari S.K., Michael N., Emerson N., Reese F., Shi Z. (2024). Spatial and single-nucleus transcriptomic analysis of genetic and sporadic forms of Alzheimer’s disease. Nat. Genet..

[24] Li Z., Martens Y.A., Ren Y., Jin Y., Sekiya H., Doss S.V., Kouri N., Castanedes-Casey M., Christensen T.A., Miller Nevalainen L.B. (2025). APOE genotype determines cell-type-specific pathological landscape of Alzheimer’s disease. Neuron.

[25] Johnson E.C.B., Dammer E.B., Duong D.M., Ping L., Zhou M., Yin L., Higginbotham L.A., Guajardo A., White B., Troncoso J.C. (2020). Large-scale proteomic analysis of Alzheimer’s disease brain and cerebrospinal fluid reveals early changes in energy metabolism associated with microglia and astrocyte activation. Nat. Med..

[26] Johnson E.C.B., Carter E.K., Dammer E.B., Duong D.M., Gerasimov E.S., Liu Y., Liu J., Betarbet R., Ping L., Yin L. (2022). Large-scale deep multi-layer analysis of Alzheimer’s disease brain reveals strong proteomic disease-related changes not observed at the RNA level. Nat. Neurosci..

[27] Johnson E.C.B., Bian S., Haque R.U., Carter E.K., Watson C.M., Gordon B.A., Ping L., Duong D.M., Epstein M.P., McDade E. (2023). Cerebrospinal fluid proteomics define the natural history of autosomal dominant Alzheimer’s disease. Nat. Med..

[28] Baik S.H., Kang S., Lee W., Choi H., Chung S., Kim J.I., Mook-Jung I. (2019). A Breakdown in Metabolic Reprogramming Causes Microglia Dysfunction in Alzheimer’s Disease. Cell Metab..

[29] West A.P., Khoury-Hanold W., Staron M., Tal M.C., Pineda C.M., Lang S.M., Bestwick M., Duguay B.A., Raimundo N., MacDuff D.A. (2015). Mitochondrial DNA stress primes the antiviral innate immune response. Nature.

[30] Tan S., Nepovimova E., Valko M., Jomova K., Wu Q., Kuca K. (2026). The cGAS-STING pathway in senescence and aging-related diseases: Mechanisms and therapeutic opportunities. Cell. Commun. Signal..

[31] Udeochu J.C., Amin S., Huang Y., Fan L., Torres E.R.S., Carling G.K., Liu B., McGurran H., Coronas-Samano G., Kauwe G. (2023). Tau activation of microglial cGAS-IFN reduces MEF2C-mediated cognitive resilience. Nat. Neurosci..

[32] Chung S., Jeong J.H., Park J.C., Han J.W., Lee Y., Kim J.I., Mook-Jung I. (2024). Blockade of STING activation alleviates microglial dysfunction and a broad spectrum of Alzheimer’s disease pathologies. Exp. Mol. Med..

[33] Thanos J.M., Campbell O.C., Cowan M.N., Bruch K.R., Moore K.A., Ennerfelt H.E., Natale N.R., Mangalmurti A., Kerur N., Lukens J.R. (2025). STING deletion protects against amyloid beta-induced Alzheimer’s disease pathogenesis. Alzheimer’s Dement..

[34] Xue F., Du H. (2021). TREM2 Mediates Microglial Anti-Inflammatory Activations in Alzheimer’s Disease: Lessons Learned from Transcriptomics. Cells.

[35] Liddelow S.A., Guttenplan K.A., Clarke L.E., Bennett F.C., Bohlen C.J., Schirmer L., Bennett M.L., Munch A.E., Chung W.S., Peterson T.C. (2017). Neurotoxic reactive astrocytes are induced by activated microglia. Nature.

[36] Guttenplan K.A., Weigel M.K., Prakash P., Wijewardhane P.R., Hasel P., Rufen-Blanchette U., Munch A.E., Blum J.A., Fine J., Neal M.C. (2021). Neurotoxic reactive astrocytes induce cell death via saturated lipids. Nature.

[37] McAlpine C.S., Park J., Griciuc A., Kim E., Choi S.H., Iwamoto Y., Kiss M.G., Christie K.A., Vinegoni C., Poller W.C. (2021). Astrocytic interleukin-3 programs microglia and limits Alzheimer’s disease. Nature.

[38] Lonnemann N., Hosseini S., Marchetti C., Skouras D.B., Stefanoni D., D’Alessandro A., Dinarello C.A., Korte M. (2020). The NLRP3 inflammasome inhibitor OLT1177 rescues cognitive impairment in a mouse model of Alzheimer’s disease. Proc. Natl. Acad. Sci. USA.

[39] Fonseca M.I., Ager R.R., Chu S.H., Yazan O., Sanderson S.D., LaFerla F.M., Taylor S.M., Woodruff T.M., Tenner A.J. (2009). Treatment with a C5aR antagonist decreases pathology and enhances behavioral performance in murine models of Alzheimer’s disease. J. Immunol..

[40] Giacomucci G., Mazzeo S., Bagnoli S., Ingannato A., Leccese D., Berti V., Padiglioni S., Galdo G., Ferrari C., Sorbi S. (2022). Plasma neurofilament light chain as a biomarker of Alzheimer’s disease in Subjective Cognitive Decline and Mild Cognitive Impairment. J. Neurol..

[41] Grande G., Valletta M., Rizzuto D., Xia X., Qiu C., Orsini N., Dale M., Andersson S., Fredolini C., Winblad B. (2025). Blood-based biomarkers of Alzheimer’s disease and incident dementia in the community. Nat. Med..

[42] Cicognola C., Janelidze S., Hertze J., Zetterberg H., Blennow K., Mattsson-Carlgren N., Hansson O. (2021). Plasma glial fibrillary acidic protein detects Alzheimer pathology and predicts future conversion to Alzheimer dementia in patients with mild cognitive impairment. Alzheimer’s Res. Ther..

[43] Suarez-Calvet M., Kleinberger G., Araque Caballero M.A., Brendel M., Rominger A., Alcolea D., Fortea J., Lleo A., Blesa R., Gispert J.D. (2016). sTREM2 cerebrospinal fluid levels are a potential biomarker for microglia activity in early-stage Alzheimer’s disease and associate with neuronal injury markers. EMBO Mol. Med..

[44] Suarez-Calvet M., Araque Caballero M.A., Kleinberger G., Bateman R.J., Fagan A.M., Morris J.C., Levin J., Danek A., Ewers M., Haass C. (2016). Early changes in CSF sTREM2 in dominantly inherited Alzheimer’s disease occur after amyloid deposition and neuronal injury. Sci. Transl. Med..

[45] Hamelin L., Lagarde J., Dorothee G., Leroy C., Labit M., Comley R.A., de Souza L.C., Corne H., Dauphinot L., Bertoux M. (2016). Early and protective microglial activation in Alzheimer’s disease: A prospective study using 18F-DPA-714 PET imaging. Brain.

[46] Friedman L.G., McKeehan N., Hara Y., Cummings J.L., Matthews D.C., Zhu J., Mohs R.C., Wang D., Hendrix S.B., Quintana M. (2021). Value-Generating Exploratory Trials in Neurodegenerative Dementias. Neurology.

[47] Butchart J., Brook L., Hopkins V., Teeling J., Puntener U., Culliford D., Sharples R., Sharif S., McFarlane B., Raybould R. (2015). Etanercept in Alzheimer disease: A randomized, placebo-controlled, double-blind, phase 2 trial. Neurology.

[48] Decourt B., Drumm-Gurnee D., Wilson J., Jacobson S., Belden C., Sirrel S., Ahmadi M., Shill H., Powell J., Walker A. (2017). Poor Safety and Tolerability Hamper Reaching a Potentially Therapeutic Dose in the Use of Thalidomide for Alzheimer’s Disease: Results from a Double-Blind, Placebo-Controlled Trial. Curr. Alzheimer Res..

[49] MacPherson K.P., Sompol P., Kannarkat G.T., Chang J., Sniffen L., Wildner M.E., Norris C.M., Tansey M.G. (2017). Peripheral administration of the soluble TNF inhibitor XPro1595 modifies brain immune cell profiles, decreases beta-amyloid plaque load, and rescues impaired long-term potentiation in 5xFAD mice. Neurobiol. Dis..

[50] Jaeger J., Staats K.A., Barnum S., Pope P., Kingery L., Buitendyk M., Cohen S., Tansey M.G., Tesi R.J., Barnum C.J. (2025). XPro1595, a Selective Soluble TNF Neutralizer, in Early Alzheimer’s Disease with Inflammation (ADi): Results from the Phase 2 MINDFuL Trial. medRxiv.

[51] ClinicalTrials.gov. A Study of XPro1595 in Patients with Early Alzheimer’s Disease with Biomarkers of Inflammation (MINDFuL). https://clinicaltrials.gov/study/NCT05318976.

[52] Xue F., Tian J., Yu C., Du H., Guo L. (2021). Type I interferon response-related microglial Mef2c deregulation at the onset of Alzheimer’s pathology in 5xFAD mice. Neurobiol. Dis..

[53] Haag S.M., Gulen M.F., Reymond L., Gibelin A., Abrami L., Decout A., Heymann M., van der Goot F.G., Turcatti G., Behrendt R. (2018). Targeting STING with covalent small-molecule inhibitors. Nature.

[54] Alector I. (2024). Alector Announces Results from INVOKE-2 Phase 2 Trial in Early AD. https://www.alzheimer-europe.org/news/alector-announces-results-invoke-2-phase-2-trial-early-ad?language_content_entity=en.

[55] ClinicalTrials.gov. A Phase I Study for Safety and Tolerability of AL002. https://clinicaltrials.gov/study/NCT03635047.

[56] Prins N.D., Harrison J.E., Chu H.M., Blackburn K., Alam J.J., Scheltens P., Investigators R.-S.S. (2021). A phase 2 double-blind placebo-controlled 24-week treatment clinical study of the p38 alpha kinase inhibitor neflamapimod in mild Alzheimer’s disease. Alzheimer’s Res. Ther..

[57] ClinicalTrials.gov. Proof-of-Concept Study of a Selective p38 Alpha Kinase Inhibitor, Neflamapimod (VX-745), in Mild Alzheimer’s Disease. https://clinicaltrials.gov/study/NCT03402659.

[58] Burns D.K., Alexander R.C., Welsh-Bohmer K.A. (2021). Safety and efficacy of pioglitazone for the delay of cognitive impairment in people at risk of Alzheimer’s disease (TOMMORROW): A phase 3, randomized, double-blind, placebo-controlled trial. Lancet Neurol..

[59] ClinicalTrials.gov. Biomarker Qualification for Risk of Mild Cognitive Impairment (MCI) Due to Alzheimer’s Disease (AD) and Safety and Efficacy Evaluation of Pioglitazone in Delaying Its Onset (TOMMORROW). https://clinicaltrials.gov/study/NCT01931566.

[60] Palmqvist S., Tideman P., Mattsson-Carlgren N., Schindler S.E., Smith R., Ossenkoppele R., Calling S., West T., Monane M., Verghese P.B. (2024). Blood Biomarkers to Detect Alzheimer Disease in Primary Care and Secondary Care. JAMA.

[61] Jack C.R., Andrews J.S., Beach T.G., Buracchio T., Dunn B., Graf A., Hansson O., Ho C., Jagust W., McDade E. (2024). Revised criteria for diagnosis and staging of Alzheimer’s disease: Alzheimer’s Association Workgroup. Alzheimer’s Dement..

[62] MahmoudianDehkordi S., Arnold M., Nho K., Ahmad S., Jia W., Xie G., Louie G., Kueider-Paisley A., Moseley M.A., Thompson J.W. (2019). Altered bile acid profile associates with cognitive impairment in Alzheimer’s disease-An emerging role for gut microbiome. Alzheimer’s Dement..

[63] Nho K., Kueider-Paisley A., MahmoudianDehkordi S., Arnold M., Risacher S.L., Louie G., Blach C., Baillie R., Han X., Kastenmuller G. (2019). Altered bile acid profile in mild cognitive impairment and Alzheimer’s disease: Relationship to neuroimaging and CSF biomarkers. Alzheimer’s Dement..

[64] Cummings J.L., Zhou Y., Van Stone A., Cammann D., Tonegawa-Kuji R., Fonseca J., Cheng F. (2025). Drug repurposing for Alzheimer’s disease and other neurodegenerative disorders. Nat. Commun..

[65] U. S. Food and Drug Administration (2025). FDA Clears First Blood Test Used in Diagnosing Alzheimer’s Disease. https://www.fda.gov/news-events/press-announcements/fda-clears-first-blood-test-used-diagnosing-alzheimers-disease.

[66] Janelidze S., Hertze J., Zetterberg H., Landqvist Waldo M., Santillo A., Blennow K., Hansson O. (2016). Cerebrospinal fluid neurogranin and YKL-40 as biomarkers of Alzheimer’s disease. Ann. Clin. Transl. Neurol..

[67] Wang Y.Y., Zhang M., Chen S.J., Miao W., Wang Z.X., Zhou Y.J., Yu S.Q., Sun Z.W., Zhou X., Yu X.F. (2025). Neuroinflammation-mediated YKL-40 correlates with tau pathology and predicts longitudinal cognitive impairment and brain atrophy in Alzheimer’s disease, with hypertensive dependency. Front. Aging Neurosci..

[68] Dubois B., López-Arrieta J., Newton R.C. (2023). Masitinib for mild-to-moderate Alzheimer’s disease: Results from a randomized, placebo-controlled, phase 3, clinical trial. Alzheimer’s Res. Ther..

[69] ClinicalTrials.gov. Masitinib in Patients with Mild to Moderate Alzheimer’s Disease. https://clinicaltrials.gov/study/NCT01872598.

[70] Reading C.L., Ahlem C.N., Murphy M.F. (2021). NM101 Phase III study of NE3107 in Alzheimer’s disease: Rationale, design and therapeutic modulation of neuroinflammation and insulin resistance. Neurodegener. Dis. Manag..

[71] ClinicalTrials.gov. A Phase 3 Study of NE3107 in Probable Alzheimer’s Disease. https://clinicaltrials.gov/study/NCT04669028.

